# Field-testing of a rapid survey method to assess the prevalence and causes of hearing loss in Gao’an, Jiangxi province, China

**DOI:** 10.1186/s13690-020-0398-1

**Published:** 2020-03-06

**Authors:** Tess Bright, Xin Shan, Jinling Xu, Jianguo Liang, Baixiang Xiao, Robbert Ensink, Islay Mactaggart, Sarah Polack, Jennifer L. Y. Yip

**Affiliations:** 1 International Centre for Evidence in Disability, London School of Hygiene & Tropical Medicine, Keppel St, London, WC1 E7HT United Kingdom; 2 Gao’an City People’s Hospital, Gao’an, Jiangxi China; 3 Zhongshan Opthalmic Centre, Sun Yatsen University, Guangzhou, Guangdong China; 4 Department of Oto-rhino-laryngology, Gelre Hospitals, Zutphen, The Netherlands

**Keywords:** China, Hearing loss, Rapid assessment, Population-based survey

## Abstract

**Background:**

The Rapid Assessment of Hearing Loss (RAHL) survey protocol aims to measure the prevalence and causes of hearing loss in a low cost and rapid manner, to inform planning of ear and hearing services. This paper reports on the first field-test of the RAHL in Gao’an County, Jiangxi Province, China. This study aimed to 1) To report on the feasibility of RAHL; 2) report on the estimated prevalence and causes of hearing loss in Gao’an.

**Methods:**

A cross-sectional population-based survey was conducted in September–October 2018. Forty-seven clusters in Gao’an County were selected using probability-proportionate-to-size sampling. Within clusters, compact segment sampling was conducted to select 30 people aged 50+. A questionnaire was completed covering sociodemographics, hearing health, and risk factors. Automated pure-tone audiometry was completed for all participants, using smartphone-based audiometry (hearTest), at 0.5, 1, 2, 4 kHz (kHz). All participants had their ears examined by an Ear Nose and Throat (ENT) doctor, using otoscopy, and probable causes of hearing loss assigned. Prevalence estimates were age and sex standardised to the Jiangxi population. Feasibility of a cluster size of 30 was examined by assessing the response rate, and the proportion of clusters completed in 1 day.

**Results:**

1344 of 1421 eligible participants completed the survey (94.6%). 100% of clusters were completed in 1 day. The survey was completed in 4.5 weeks. The prevalence of moderate or greater hearing loss (pure-tone average of 0.5, 1, 2, 4 kHz of > = 41dBHL in the better ear) was 16.3% (95% CI = 14.3, 18.5) and for any level of hearing loss (pure-tone average of > = 26dBHL in the better ear) the prevalence was 53.2% (95% CI = 49.2, 57.1). The majority of hearing loss was due to acquired sensorineural causes (91.7% left; 92.1% right). Overall 54.0% of the population aged 50+ (108,000 people) are in need of diagnostic audiology services, 3.4% were in need of wax removal (7000 people), and 4.8% were in need of surgical services (9500 people). Hearing aid coverage was 0.4%.

**Conclusion:**

The RAHL survey protocol is feasible, demonstrated through the number of people examined per day, and the high response rate. The survey was completed in a much shorter period than previous all-age surveys in China. Some remaining challenges included assignment of causes of probable sensorineural loss. The data obtained from this survey can be used to scale-up hearing services in Gao’an.

## Background

In 2018, the World Health Organization (WHO) estimated that 466 million people globally had disabling (moderate or greater) hearing loss - 106 million more than the previous estimate from 2012 [1]. The higher estimates are explained by the increase in global population since 2012 from 7.1 billion to 7.6 billion, as well as global ageing [2]. The majority of individuals (> 80%) who experience hearing loss live in low and middle income countries (LMICs) [1]. This is partly because of the distribution of the world’s population, however there is also evidence that the prevalence of hearing loss is greater in LMICs than in high-income countries. For instance in South Asia, the age-standardised prevalence amongst adults is estimated to be 17.0% vs 4.9% in high-income regions [3]. This inequitable distribution may be explained by the increased exposures to risk factors across the life course in LMICs, compared to high income countries [4]. For example, increased exposure to infectious diseases, unregulated use of ototoxic medications, and noise exposure in the workplace [5]. The WHO estimate that over a third of people with moderate or greater hearing loss are aged over 65 years [6].

Global estimates must be interpreted in light of the very limited data on population prevalence, and causes of hearing loss, particularly from LMICs. Evidence used to calculate current global estimates includes less than 50 surveys of hearing loss, the first conducted in 1982 and most recent in 2006. The most recent survey in sub-Saharan Africa was conducted in 2003, more than 15 years ago. Out of the 46 countries in sub-Saharan Africa, 11 surveys of hearing loss have been conducted (i.e. less than a quarter of countries), and only eight of these were all-age population-based surveys. In China, a country of over 1.3 billion people, one previous all-age survey has been conducted, with a small number of others focusing on particular age groups (elderly, children) [7, 8]. These gaps in data result in substantial uncertainty around global estimates [9]. Arguably, global estimates have limited value for decisions around health service provision at a country or district level [10]. Much more useful for this purpose is locally derived epidemiological data [10].

The substantial impact of hearing loss on communication, speech and language development has been well-described [11]. Hearing loss is associated with poor educational attainment, reduced employment opportunities and poverty [12–14]. There is also a growing body of evidence linking hearing loss to the development of depression, dementia and other mental health conditions among adults [15–17]. Hearing loss is also linked with poor quality of life [18]. This evidence provides an important rationale for efforts to ensure timely access to affordable services of high quality. The Sustainable Development Goals (SDGs) are a set of goals and targets developed and agreed upon by the United Nations in 2015. A central component of the health-specific goal is Universal Health Coverage (UHC), which aims to “ensure all people have access to needed health services including prevention, promotion, treatment, rehabilitation, and palliation, of sufficient quality to be effective whilst also ensuring that those who use the services are not exposed to financial hardship”. Thus, given the substantial number of people with hearing loss, without good access to ear and hearing services, UHC will not be achieved. Evidence suggests that the current level of access to hearing specific services is inadequate in LMICs [19, 20]. A recent systematic review of access to rehabilitation for people with disabilities found that coverage of assistive devices for people with hearing loss was unacceptably low across LMICs where data existed, ranging from 0 to 24% at the population-level [21].

Previous research has described the difficulties with conducting all-age population-based surveys of hearing loss, which includes high cost equipment, lack of human resources, large sample sizes required and lack of global attention [3, 22, 23]. The field of visual impairment and blindness has substantially more prevalence data (> 300 surveys) which has been made possible through rapid assessment protocols such as the Rapid Assessment of Avoidable Blindness (RAAB). In order to facilitate increased data collection on hearing loss, an equivalent protocol – the Rapid Assessment of Hearing Loss (RAHL) has been developed. This survey methodology uses a standardised sampling procedure, simplified examination protocol, and focusses on people aged 50 + [23]. This methodology is lower cost than a full all-age epidemiological survey, through the smaller required sample size (prevalence in those aged 50+ is higher), and lower cost equipment. The aim of the RAHL surveys are to estimate the prevalence of hearing loss, probable causes, and current level of service coverage in order to inform locally-appropriate service planning. The RAHL methodology has been piloted in Malawi to refine elements of the protocol including the cluster size, and the questionnaires. This paper reports the first field-test of the RAHL methodology among adults age 50+ in Gao’an district, China.

The main purposes of this study were:

1. To report on the feasibility of conducting the RAHL survey
2. To estimate the prevalence and causes of hearing loss amongst people aged 50+ in the population of Gao’an, China.

## Methods

### Development of RAHL methodology

The RAHL methodology has been developed through an iterative process. Firstly, we conducted a secondary data analysis of previous all-age population-based surveys conducted in India [24] and Cameroon [25]. This analysis found that the majority (> 70%) of hearing loss was experienced by people aged 50+, and the distribution of causes in this age group were representative of the total population [23]. Secondly, a review of the literature was conducted to determine the most appropriate clinical tools to use in a survey of older people to assess hearing levels and assign causes. This drew on the previously developed WHO protocol for conducting all-age population-based surveys of hearing loss [26]. Thirdly, a questionnaire, including a clinical assessment protocol was developed through a literature review and expert consultation. This included the development of an algorithm for assigning causes related to the outer and middle ear. Then, a pilot study of the survey protocol was undertaken in Malawi to determine the appropriate cluster size, and whether the data collection protocol needed adaptations. Finally, a clinic-based diagnostic accuracy study was undertaken in Malawi to determine whether non-specialist health workers, instead of highly skilled professionals, could undertake the hearing test and ear examination [27]. The next section gives an overview of the developed methodology.

### Study design

The RAHL is a population-based cross-sectional household survey. The target population is people aged 50 + .

### Sample size and sampling

The sample size is calculated based on the expected prevalence of moderate or greater hearing loss in people aged 50+, desired confidence of 95%, 20% precision around the estimate, a design effect (DEFF) of 1.5 (conservative estimate based on data from India and Cameroon), and response rate of 90% [23].

A two-stage sampling procedure is used to select firstly clusters, and then individuals within selected clusters. This methodology has been widely used in the RAAB survey developed by the International Centre for Eye Health at the London School of Hygiene & Tropical Medicine [28]. In the first stage, clusters, usually the smallest census enumeration area, are selected via probability-proportionate-to-size sampling – using the most recent census as a sampling frame. In the second stage, households within clusters are selected using compact segment sampling, whereby clusters are divided in to segments containing approximately 30 people aged 50 years and older. The segmentation is usually done using maps of the selected clusters. Maps can be obtained in a range of ways - drawn by a village leader, provided by the national statistics office, or using online tools (e.g. Google Maps). A cluster size of 30 was determined based on a pilot of the methodology in Malawi. All people aged 50 years and older who have been living in the selected household at least 6 months of the previous year are considered eligible for inclusion in the survey.

### Data collection teams

Based on the pilot in Malawi, the team makeup for the RAHL includes:

- **One enumerator**: to enumerate eligible participants, obtain informed consent, and complete a sociodemographic questionnaire
- **Two hearing testers:** to complete hearing screening. The pilot in Malawi found that with only one hearing tester per team, less than 20 people can be seen per day. In order to reach an adequate cluster size (i.e. at least 30 people), two testers were required. The diagnostic accuracy study in Malawi found that non-specialist health workers can be trained to accurately assess hearing [27].
- **One ear examiner:** to complete an ear examination using otoscopy and assign causes (Ear Nose and Throat (ENT) specialist). Pilot work in Malawi suggested at least an ENT clinical officer (mid-cadre health worker with 18 months of clinical training) is required to be in the field [27].

All team members are trained for at least 5 days prior to undertaking the RAHL on study procedures, ethical considerations, and clinical tests. This training includes an inter-observer variation (IOV) study to check consistency in diagnosis across assessors.

### Data collection procedures

In households with eligible individuals, a paper-based household roster is completed, recording basic information about eligible members of the household. Mobile-based data collection, using the platform Open Data Kit (ODK), is used to collect questionnaire data. Data collection includes the following steps:

1. All participants complete a general questionnaire covering demographics, household characteristics and assets-ownership, self-reported hearing loss, and risk factors for hearing loss.
2. Each participant has their hearing screened by a trained person using a validated mobile-based automated audiometry system, hearTest (hearX group) [29, 30]. Thresholds are obtained at 0.5, 1, 2, and 4 kHz in each ear. Prior to fieldwork, the equipment is calibrated to audiological standards. Environmental noise is often a problem in field-surveys, hearTest is paired with low cost over the ear headphones (Sennheiser HD280) that provide additional attenuation to help overcome problems with background noise [31].
3. All participants have their ears examined using otoscopy by an ENT specialist or equivalent and diagnosis made. Outcomes include normal ear examination, otitis externa (OE), impacted wax (IW), foreign body (FB), acute otitis media (AOM), otitis media with effusion (OME), chronic otitis media (COM) – wet perforation, COM – dry perforation, and other middle ear condition.
4. Probable causes of hearing loss assigned for those with hearing loss. Outcomes include OE, IW, FB, AOM, OME, COM – dry, COM – wet, other middle ear, acquired sensorineural, congenital sensorineural, or mixed.
5. Those with any level of hearing loss or ear disease are asked about previous care seeking for the condition.
6. Simple conditions (e.g. wax removal) are treated in the field, and more complex conditions are referred to the nearest services which are identified in advance of the survey.

#### Assigning the probable causes of hearing loss

Assigning the exact cause of hearing loss is difficult, even in high-resource settings, with all available clinical tools. There is always a trade-off between the level of accuracy and costs. The more detail needed, the higher the survey costs. Given the complexities with assigning a cause the ear examiner can only assign the “probable” causes. This survey protocol takes a pragmatic approach that is feasible in the field, allowing appropriate conclusions to be drawn about what the key service needs are for the population.

To help standardise the assignment of probable causes related to the middle and outer ear (COM - wet, COM -dry, OME, AOM, OE, IW, FB), an algorithm was developed using a review of the literature, expert consultation, and a pilot-test in a clinic-based study in Malawi (see Appendix 1). The algorithm is programmed in to the ODK form as a “decision support system” to guide the examiner. Once the examiner has filled out the questionnaire items related to the features of the ear (e.g. colour of ear drum, presence of discharge, pain etc), a prompt appears in the form with a suggested cause. If the examiner does not agree with the diagnosis, they are required to specify the reason for this. This system allows for clinical judgement, whilst also standardising the assignment of common middle ear causes.

For causes related to the inner ear (i.e. congenital, noise-induced, ototoxic medication, non-infectious disease, infectious disease, or unknown), the assignment is based on clinical history of hearing loss and risk factors (obtained from the questionnaire), the results of the ear examination, and hearing test. Sensorineural causes are grouped in to acquired or sensorineural.

#### Assigning the type of hearing loss

Causes are grouped in to broad type categories, either probable conductive, sensorineural, or mixed. If the ear examination is abnormal and hearing loss present, it is assumed that hearing loss is conductive. If the ear examination is normal and hearing loss is present, it is assumed that the hearing loss is sensorineural. A decision support system is programmed in to the ODK questionnaire to prompt the clinician on the probable type. This is based on the results of the ear examination. At this stage, the clinician can agree or disagree with the suggested type, and if they disagree, a reason for this specified.

#### Service needs

Table 1 outlines the the likely service needs (diagnostic, surgical, medical) according to the diagnosis. As shown in the table, the service needs for ear disease varies, but for sensorineural or mixed hearing loss the service need is for further diagnostic assessment, and potential hearing aid fitting. The RAHL considers that outer and middle ear pathologies require services regardless of whether hearing loss is present. Diagnostic audiological assessment is needed when a bilateral hearing loss is present which is sensorineural (acquired or congenital) or mixed in nature in both ears. A person may have more than one service need based on the diagnosis in each ear.

**Table 1:** Service needs according to diagnosis

Need	Diagnosis	Hearing test result	Description
Diagnostic audiology assessment (possible hearing aid)	Sensorineural (acquired or congenital) or mixed hearing loss in both ears	Bilateral hearing loss (> 25 dB HL)	This involves air and bone conduction audiometry in sound proof room to confirm hearing levels and potential hearing aid fitting (depending on degree, and duration of loss). If a hearing aid is not suitable, other interventions such as cochlear implants, or sign language training may be suitable [32]. For mixed hearing loss, the underlying cause of the conductive component also needs to be treated.
Surgical assessment	Chronic otitis media – wet perforation Chronic otitis media – dry perforation Chronic otitis media – possible cholesteatoma	N/A^a^	Chronic otitis media – wet or dry require tympanoplasty to mend tympanic membrane [33] Chronic otitis media – possible cholesteatoma requires surgery to remove
Medication	Acute otitis media (AOM) Chronic otitis media – wet perforation Chronic otitis media – possible cholesteatoma Otitis externa (OE)	N/A^a^	AOM requires analgesics, and antibiotics [34] Chronic otitis media – wet perforation requires aural toilet, and ear drops (medication) to allow otorrhea (discharge) to clear prior to surgery [33]. Chronic otitis media – cholesteatoma may also require medication OE requires antifungal or antibiotic ear drops [35]
Impacted wax or foreign body removal	Impacted wax (IW) Foreign body (FB)	Hearing loss > 25 dB HL in either ear	IW and FB are removed using suction or hooks depending on skill and equipment availability [51]
Review (“watchful waiting”)	Otitis media with effusion (OME)	Hearing loss > 25 dB HL in either ear	OME requires re-examination of ears, and repeat audiometry [37]

^a^Need for service does not depend on whether hearing loss is present

Hearing aid coverage was estimated using the following calculation:

$$ HAC=\frac{a}{\left(a+b\right)}\ast 100 $$

Where,

- *a* is the number of participants with any level of hearing loss (bilateral), probable mixed or sensorineural in nature in both ears, who report that they own a hearing aid (met need);
- *b* is the number of people with any level of hearing loss (bilateral), probable mixed or sensorineural in nature in both ears, who report not owning a hearing aid (unmet need)

We recognise that some people with conductive hearing loss may benefit from hearing aids, however we assume the medical or surgical intervention would precede the rehabilitation.

### Study outcomes

The key RAHL study outcomes include the following:

1. **Prevalence of hearing loss:** The WHO definitions of hearing loss are used, which are based on pure-tone average of 0.5, 1, 2, and 4 kHz in the better ear. Estimates are obtained for: “disabling hearing loss” referred to in this paper as moderate or greater hearing loss (> = 41dBHL), any level of hearing loss (> = 26dBHL), and degree of hearing loss (mild 26-40dBHL; moderate 41-60dBHL; severe 61-80dBHL; and profound 81dBHL or greater) [38, 39].
2. **Prevalence of ear disease:** The outcome of the ear examination was analysed to obtain the prevalence of ear disease, regardless of the presence of hearing loss.
3. **Probable causes of hearing loss:** in each ear amongst those with any level of hearing loss. The options for the causes of hearing loss are provided in Table 1.
4. **Need and coverage of services:** based on the diagnoses made (see Table 1). Hearing aid coverage calculated based on the definition above.
5. **Access and barriers:** For those with hearing loss or ear disease identified, previous care seeking, and barriers to accessing care.
6. **Associations:** between hearing loss and risk factors. This is expected to be important for informing prevention strategies, and may help to overcome the previous challenges identified in field surveys where a large proportion of causes are unknown [20, 22, 40].

### Field-testing the RAHL in Gao’an, China

#### Setting, sample size and sampling

Field testing of the RAHL was conducted in Gao’an, Jiangxi province, China in September–October 2018. Gao’an is a county-level rural city, covering an area of 2430 km^2^, comprised of 20 towns and 365 villages with a population of approximately 1 million people. One public hospital (Gao’an People’s Hospital) provides ear and hearing care services. Hearing aid fittings, and surgeries require referrals to Nanchang, the capital city of Jiangxi province (one-hour drive from Gao’an). The research was conducted in collaboration with Gao’an People’s Hospital by two teams.

The sample size for this setting was determined to be 1412 based on a conservative expected prevalence of 10%. Thus 47 clusters were required to meet the desired sample size. Clusters (villages) were selected from the 2017 Jiangxi census (sampling frame), using probability-proportionate-to-size sampling.

Obtaining maps for segmentation proved difficult. The villages, particularly in urban areas were densely populated, with most people living in apartments in multi-story buildings. Instead of mapping, lists of “natural villages”, (sub-villages) and their population sizes were obtained from community leaders. These natural villages were numbered, and one area randomly selected as the segment using a random number generator. Each sub-village was approximately the same size as our desired segment size. If the selected sub-village was too large, it was further segmented on arrival in the segment with the assistance of a community leader. If it was too small, it was combined with a neighbouring sub-village. Community sensitisation was conducted in advance of the survey to assist in maximising response rates. Within clusters, community leaders assisted with sampling, and identification of eligible individuals.

Each team included:

- One enumerator: medical student
- Two hearing testers: one audiometry nurse; one medical student
- One ear examiner: ENT specialist.

The IOV study revealed 100% agreement between results obtained on hearing tests across clinicians, using the most experienced audiology nurse as a gold standard. For ear examination the agreement was 85.7% in both the left and rights ears.

### Data analysis and outcome variables

All analysis was conducted in Stata 15.0 (StataCorp LP, College Station, Texas).

#### Feasibility outcomes

Feasibility of a cluster size of 30 was examined by assessing the response rate, proportion of clusters completed in 1 day, number of survey days, and missing data.

The time taken to complete the hearing test was recorded within the hearTest application. To assess the reliability of hearTest, three factors were considered: 1) 1 kHz test-retest reliability (1 kHz assessed twice in each ear); 2) false response rate (measured in hearTest); 3) ambient noise levels compared to the headphone maximum permissible ambient noise levels (MPANLs).

Shapiro-Wilk tests were conducted to assess normality of time, and false response data. Based on evidence of non-normally distributed data, medians and interquartile ranges (IQR) were obtained to summarise time data.

#### RAHL outcomes

To account for clustering survey design the “svy” command was employed. To account for non-response according to age and sex, weighting was applied to the prevalence estimates to ensure they were representative of the population. This was conducted using the 2010 census data age and sex demographics of Jiangxi province, with five-year age stratum. Strata were compared to the observed population distribution in the sample, and weights calculated in the following manner:

weight of stratum x = (population in stratum x * total sample size)/(total population * sample size in stratum x).

The derived weights were applied to estimate prevalence of hearing loss and ear disease. 95% confidence intervals (CI) were obtained around all estimates. Prevalence estimates were disaggregated by age and sex.

 The probable causes of any level of hearing loss were determined in each ear, and grouped by probable type. Consistency in the assignment of type was checked. For example, if impacted wax was assigned as the cause, but the degree of hearing loss was severe or greater, then the type was reassigned as mixed. The need for services was calculated based on Table 1. Need for wax or foreign body removal, medication, and surgery were calculated at the level of the person, and in terms of the number of ears. Diagnostic audiology was only calculated at the person-level given the requirement for bilateral hearing loss. The needs for services were extrapolated to the population of Gao’an. Univariate logistic regression was performed to examine factors associated with hearing loss in the population. The dependent variable was mild or greater hearing loss (binary). Exposure variables included age, sex, literacy, socioeconomic position, and self-reported risk factors for hearing loss (e.g. noise exposure, ototoxic medication, history of infectious diseases, head trauma, smoking, diabetes, high blood pressure). Age and sex were considered as a priori confounders. Multivariate analysis was conducted, including exposures that were significant on the univariate analysis. Poverty indicators (household construction materials, asset ownership) were used to derive an index for socioeconomic position (SEP), using principal components analysis (PCA) to generate quartiles. The poverty indicators were based on previous household surveys in China and consultation with team members during training [41, 42, 52].

## Results

### Feasibility of the survey protocol

Of 1421 eligible participants, 1344 people were examined (94.6%), 38 were not available (2.7%) and 39 refused (2.7%). The survey took 4.5 weeks (24 days) to complete with four teams. All 47 clusters were completed by one team in 1 day. The response rate for the survey was very high (94.6%). Of 1344 participants, 1342 completed the hearing test – with two missing tests due to illnesses (e.g. dementia). There were no other missing examinations or questionnaires. The median duration of the hearing test was 7.7 min (IQR 6.9, 8.7). Reliability of testing was high with over 90% of test-retests at 1000 Hz within +/− 5 dB in both ears. Nearly all of tests had a false response rate of 10% or less (85.7%). The median false response rate was 4%. Ambient noise did not appear to impact testing in this study, the vast majority of tests were conducted in areas where ambient noise did not exceed the MPANLs (see Appendix 3).

### Overview of the study population

Compared with census data, there was an underrepresentation of men, particularly in the younger age groups (Table 2). A more detailed comparison between the census and the sample can be found in Appendix 2.

**Table 2:** Characteristics of study subjects by examination status, age group and sex (comparing to population of Jiangxi province, 2010 census data), n (%)

	Population in Jiangxi	Available	Not available	Refused	Total
Overall 50+	9,868,486 (100.0)	1344 (94.6)	38 (2.7)	39 (2.7)	1421 (100.0)
Male	4,933,243 (50.0)	533 (39.7)	28 (71.8)	22 (57.9)	583 (39.7)
Female	4,935,243 (50.0)	811 (60.3)	11 (28.2)	16 (42.1)	838 (71.8)
Age group					
50–59	4,769,228 (48.3)	408 (30.4)	25 (64.1)	11 (29.0)	444 (31.3)
60–69	2,854,726 (28.9)	522 (38.8)	10 (25.6)	19 (50.0)	551 (38.8)
70–79	1,659,973 (16.8)	297 (22.1)	3 (7.7)	5 (13.2)	305 (21.5)
80–89	532,685 (5.4)	104 (7.7)	1 (2.6)	2 (5.3)	107 (7.5)
90+	51,874 (0.5)	13 (1.0)	0 (0.0)	1 (2.6)	14 (1.0)

### Prevalence of hearing loss and ear disease

The prevalence of moderate or greater hearing loss in the 50+ population of Gao’an was 16.3% (95% CI = 14.3, 18.5) and for any level of hearing loss 53.2% (95% CI = 49.2, 57.1) (Table 3). An increase in prevalence of moderate or greater and any hearing loss was seen with age, for both males and females. The prevalence of moderate or greater hearing loss in increased from 5.2% (95% CI = 3.0, 8.8) in those aged 50–59 years to 64.9% (95% CI = 54.4, 74.1) in those aged 80+ years. No significant differences were seen in prevalence by sex, across age groups and by degree. The prevalence decreased with increasing severity – from 36.9% (95% CI = 33.3, 40.7) with mild hearing loss, to 0.6% (95% CI = 0.3, 1.0) with profound loss. This pattern was also seen within age groups, regardless of sex.

**Table 3:** Distribution of the prevalence of hearing loss by degree, and sex (adjusted for age and sex of total population) amongst 1344 people in Gao’an, Jiangxi, China

	All		Male		Female	
	N	% (95% CI)	N	% (95% CI)	N	% (95% CI)
Moderate or greater hearing loss (> 40 dB better ear)						
All	283	16.3 (14.3, 18.5)	125	16.2 (13.4,19.4)	158	16.4 (13.8, 19.5)
50–59	20	5.2 (3.0, 8.8)	7	5.5 (2.5, 11.5)	13	4.8 (2.6, 8.8)
60–69	83	14.8 (11.7, 18.5)	41	16.4 (11.6, 22.6)	42	13.1 (9.9, 17.1)
70–79	101	34.5 (28.6, 41.0)	51	36.3 (29.0, 44.2)	50	32.9 (25.5, 41.3)
80+	79	64.9 (54.4, 74.1)	26	59.7 (36.2, 79.5)	53	68.2 (58.1, 76.8)
Any level (> 25 dB ear better ear)						
All	789	53.2 (49.2, 57.1)	339	55.6 (49.9, 61.2)	450	50.7 (46.6, 54.8)
50–59 years	137	36.5 (30.3, 43.1)	55	42.7 (33.0, 52.9)	82	30.1 (24.5, 36.4)
60–69 years	300	55.9 (50.8, 60.7)	136	58.2 (50.6, 65.5)	164	53.4 (47.6, 59.0)
70–79 years	236	81.2 (75.4, 85.9)	109	78.9 (70.3, 85.5)	127	83.3 (75.3, 89.1)
80+ years	116	98.7 (91.4, 99.8)	77	96.7 (79.5, 99.6)	39	100.0
Degree (better ear)						
None (0-25 dB)	553	46.8 (42.9, 50.7)	192	44.3 (38.7,50.0)	361	49.3 (45.2, 53.4)
Mild (26-40 dB)	506	36.9 (33.3, 40.7)	214	39.5 (33.9, 45.5)	292	34.3 (30.9, 37.9)
Moderate (41-60 dB)	201	11.7 (10.1, 13.7)	109	11.5 (9.4, 13.9)	92	12.0 (9.5, 15.2)
Severe (61–80 dB)	70	4.0 (3.0, 5.3)	43	4.4 (3.1, 6.2)	27	3.5 (2.3, 5.3)
Profound (81 dB +)	12	0.6 (0.3, 1.0)	6	0.5 (0.2, 1.2)	6	0.6 (0.3, 1.4)

The prevalence of any ear disease was 6.0% (95% CI = 4.7, 7.5) in the left and 6.0% (95%CI = 4.5, 8.0) in the right. Overall, 6.0% (95% CI = 4.7, 7.5) of ears had ear disease. The most common type of ear disease was impacted wax (left 2.3% (95% CI = 1.6, 3.2); right 2.0% (95% CI = 1.3, 3.1). The majority of participants had a normal ear examination (left 93.8% (95% CI = 92.1, 95.1); right 93.7% (95% CI = 91.7, 95.3) (Table 4).

**Table 4:** Age and sex adjusted prevalence ear disease in the population (with or without hearing loss)

	Left ear		Right ear		Total ears	
	N	% (95% CI)	N	%	N	%
Any ear disease	86	6.0 (4.7, 7.5)	80	6.0 (4.5, 8.0)	166	6.0 (4.7, 7.5)
Outer						
Impacted wax	37	2.3 (1.6, 3.2)	33	2.0 (1.3, 3.1)	70	2.2 (1.6, 3.0)
Otitis externa	5	0.7 (0.3, 1.8)	3	0.6 (0.2, 1.7)	8	0.6 (0.3, 1.6)
Middle						
COM – wet perforation	12	1.0 (0.5, 2.0)	11	0.9 (0.4, 1.7)	23	0.9 (0.5, 1.6)
COM – dry perforation	26	1.6 (1.0, 2.5)	24	1.7 (1.1, 2.8)	50	1.7 (1.1, 2.5)
OME	1	0.07 (0.01,0.5)	5	0.4 (0.1, 1.1)	6	0.2 (0.07, 0.6)
Other middle ear	5	0.3 (0.1, 0.7)	4	0.4 (0.1, 1.2)	9	0.3 (0.2, 0.7)
Normal ear examination	1255	93.8 (92.1, 95.1)	1261	93.7 (91.7, 95.3)	2516	93.8 (92.1, 95.0)
*Total*	1344^a^	100.0	1344^a^	100.0		2688 (100.0)

^a^3 participants missing ear examination

### Probable causes of hearing loss

The main probable cause of hearing loss (any level) was acquired sensorineural hearing loss (91.7% left; 92.1% right) followed by COM (dry perforation) (1.5% left; 1.3% right) and impacted wax (2.2% left; 2.3% right) (Table 5). Less than 5% of the probable causes were probable conductive in nature. In the right ear, a similar pattern was observed. Close to 3% of hearing loss was probable mixed in nature in both ears, with the main conductive component cause by impacted wax and dry perforations.

**Table 5:** Probable causes and type of hearing loss (by ear) amongst those with any level of hearing loss (> 25 dB in better ear)

	Left ear (*n* = 789)		Right ear (*n* = 789)	
	N	%	N	%
Probable conductive				
Impacted wax	17	2.2	18	2.3
Otitis externa	3	0.4	2	0.3
COM – wet perforation	5	0.6	5	0.6
COM – dry perforation	12	1.5	10	1.3
OME	0	0.0	3	0.4
Other middle ear	2	0.3	0	0.0
*Total*	39	4.9	38	4.8
Probable sensorineural				
Acquired	723	91.6	727	92.1
Congenital	1	0.1	0	0.0
*Total*	724	91.7	727	92.1
Probable mixed				
Impacted wax	11	1.4	8	1.0
Otitis externa	0	0.0	2	0.3
COM – wet perforation	3	0.4	2	0.3
COM – dry perforation	11	1.4	10	1.3
OME	1	0.1	1	0.1
Other	0	0.0	1	0.1
*Total*	26	3.3	24	3.0

### Population needs and coverage of services

Over half of participants needed diagnostic audiology and possible hearing aid fitting (54.0%). Extrapolated to the total population of Gao’an, this represents 108,000 people aged 50+. Wax removal services were required for 3.4% of the population (7000 people). Surgical assessments were required for 4.8% (9500 people). Medication was required for 2.0% of the population (4000 people). (Table 6).

**Table 6:** Needs for services in the population of Gao’an, Jiangxi Province, China

		People in need			Ears		
		Sample (*n* = 1344)		Population^a^	Sample (*n* = 1344*2)		Population^a^
	Definition of need	N	%	n	N	%	n
Diagnostic audiology (possible hearing aid)	Bilateral sensorineural or mixed type of hearing loss (>25dB)	726	54.0	108,000	-	-	-
Surgery	COM (any) with or without hearing loss	65	4.8	9,500	75	2.8	11,000
Medication	AOM, OE, COM (wet) with or without hearing loss	27	2.0	4,000	32	1.2	5,000
Wax or foreign body removal removal	Impacted wax with hearing loss (>25 dB) in either ear	46	3.4	7,000	59	2.2	9,000
Watchful waiting	OME; Hearing loss >25dB HL in either ear	4	0.5	500	5	0.2	500

^a^based on 200,000 people aged 50+ (20% of 1 million population); rounded to nearest 500; ^*^number of ears

Of those in potential need of hearing aids, only three reported that they had hearing aids (0.4% coverage). Four additional people also reported wearing hearing aids who were in need of surgical services for COM. Of those in need who owned hearing aids, a third (33.3%; *n* = 1) reported that they “seldom” wore them, the remaining two reporting that they always wore them. Reasons for this included “too much trouble to wear”, and “sound too loud”. Of those with hearing loss in either ear and/or ear disease in either ear, only 5.3% had previously sought care. Of these, 83.6% sought care at the public hospital, and 80% received medication. The vast majority of people (97.8%) who had not sought care who had hearing loss or ear disease said that they did not feel the need to go for services.

### Risk factor analysis

The most commonly reported health condition known to be associated with any level hearing loss was hypertension (28.8%), followed by diabetes (8.6%) and smoking (15.3% every day; 3.1% some days) (Table 7). Other risk factors were less common. In univariate regression, hearing loss (any level) increased with age with 12% increase in odds of hearing loss for each year (OR = 1.12; 95% CI = 1.10, 1.14). Females had 0.73 times lower odds of hearing loss than males. People were literate had lower odds of hearing loss. History of malaria was associated with an increased odds of hearing loss. These factors remained significant in multivariate analysis. No other significant associations were observed.

**Table 7:** Association between any level of hearing loss and risk factors

	N (%)	Univariate		Multivariate	
		aOR^ (95% CI)	*P* value	aOR^b^ (95% CI)	*P* value
Age (continuous)	–	1.12 (1.10, 1.14)	**< 0.001**	1.11 (1.09, 1.13)	**< 0.001**
Sex					
Male	533 (39.6)	1.0 (base)	–	1.0 (base)	–
Female	811 (60.3)	0.73 (0.55, 0.97)	**0.029**	0.68 (0.50, 0.93)	**0.016**
Highest education					
Less than primary	347 (25.8)	1.0 (base)	**–**		
Primary	701 (52.2)	0.70 (0.48, 1.02)	0.061	–	–
Secondary	270 (20.1)	0.80 (0.50, 1.29)	0.354	–	–
Post-secondary	26 (1.9)	0.56 (0.14, 1.51)	0.194	–	–
Literacy					
Unable to read	358 (26.6)	1.0 (base)	–	1.0 (base)	–
Able to read	986 (73.4)	0.71 (0.50, 0.99)	**0.045**	0.71 (0.50, 0.99)	**0.048**
SEP quintile					
1 - poorest	304 (22.7)	1.0 (base)	–	–	–
2	366 (27.3)	0.84 (0.54, 1.32)	0.670	–	–
3	336 (25.0)	0.92 (0.55, 1.53)	0.742	–	–
4 - richest	336 (25.0)	0.77 (0.45, 1.31)	0.321	–	–
Noise exposure	18 (1.3)	1.05 (0.35, 3.13)	0.924	–	–
Hypertension	387 (28.8)	0.88 (0.63, 1.23)	0.451	–	–
Diabetes	115 (8.6)	0.92 (0.57, 1.46)	0.707	–	–
Cancer medication	20 (1.5)	2.68 (0.82, 8.72)	0.099	–	–
Trauma	53 (3.9)	1.05 (0.54, 2.06)	0.880	–	–
Smoking					
Not at all	1097 (81.6)	1.0 (base)	–	–	–
Some days	41 (3.1)	0.73 (0.25, 2.10)	0.549	–	–
Every day	206 (15.3)	1.33 (0.84, 2.10)	0.217	–	–
HIV	0 (0.0)	–	–	–	–
Malaria	76 (5.7)	1.68 (1.04, 2.70)	**0.034**	1.67 (1.08, 2.59)	**0.022**
TB	11 (0.8)	1.04 (0.31, 3.46)	0.954	–	–
Other infectious disease^a^	52 (3.8)	1.13 (0.51, 2.49)	0.762	–	–

*SEP* Socioeconomic position, *TB* Tuberculosis; ^a^meningitis, chicken pox, pneumonia, herpes zoster, syphilis, mumps, measles, *aOR* Adjusted odds ratio; ^adjusted for age and sex; ^b^adjusting for all other variables in the model.

## Discussion

### Feasibility of RAHL methodology

This study describes the development and field-testing of the RAHL methodology for assessing the prevalence of hearing loss in population-based surveys in Gao’an, Jiangxi, China. The survey methodology focuses on people aged 50+ based on evidence from previous surveys that the majority of hearing loss is experienced by this age group [23]. Mobile-based data collection, as well as validated smartphone hearing testing (hearTest) were utilised.

The survey took 4.5 weeks (24 days) to complete with four teams, for a sample size of 1344. In comparison to an all-age survey conducted in 2005–2006, which took 20 months to complete four provinces, and the 60+ survey in 2014–2015 which took 13 months, this is rapid and therefore likely far lower cost [8, 43]. We used a rigorous sampling method to select participants, which worked well in practice. Villages in Gao’an were relatively dense, which did not require teams to walk great distances to identify eligible participants. Community sensitisation in advance of the survey, and working with local leaders and health workers on the day of the survey facilitated high response rates (> 90%). This meant that teams did not need to make repeat visits to villages – 100% of clusters were completed in 1 day by one team. House-to-house sampling also assisted with achieving this high response rate, rather than testing at a central location, as is recommended in the previous WHO Ear and Hearing Disorders Survey protocol [26, 44]. These findings reinforce findings from the pilot in Malawi that a cluster size of 30 is feasible for the survey. There was very limited missing data, highlighting the benefits of using mobile-based data collection.

Considering the assessment protocol, using smartphone-based automated audiometry (hearTest) was well-accepted by participants. Using hearTest facilitated house to house sampling, without the need for a power source. The over the ear headphones used with hearTest as well as testing inside participant homes assisted with reducing background noise, which was not a significant problem in this survey. Only two people could not be tested, both due to other health conditions. Test took approximately 7 min per person, and reliability was high. In further planned field tests of the RAHL protocol, the exact time for each component of the survey will be measured to allow more detailed planning.

### RAHL survey outcomes

The prevalence of moderate or greater and any level of hearing loss were estimated to be 16.3% (95% CI = 14.3, 18.5) and 53.2% (95% = 49.2, 57.1) respectively. There was no difference in prevalence by sex, but prevalence increased with age. The prevalence decreased with severity of hearing loss from 36.9% (95%CI = 33.3, 40.7) mild, to 0.6% (95% CI = 0.3, 1.0) profound. Overall, 6.0% (95% CI = 4.7, 7.5) of ears had ear disease.

In both the left and right ear, the main probable cause was acquired sensorineural hearing loss (91.7% left; 92.1% right). Age, sex, and malaria were associated with increased risk of hearing loss. Literacy, used as a proxy measure for higher SEP, had a protective effect. Females had a decreased risk of hearing loss compared to males.

Compared to previous studies, in 2005–2006, an all-age prevalence study was conducted in Jiangsu, Sichuan, Guizhou and Jilin provinces in China and found an all-age prevalence of moderate or greater hearing loss of 4.4% and a prevalence of 54.5% in people aged 50 + [43]. These findings concur with the findings in our study. A more recent population-based survey (2014–2015) of 6984 adults in Jilin, Guangdong, Shaaxi and Gansu found a prevalence of any level of hearing loss of 58.9% in people aged 60+. This is also comparable to our estimate for any-level of hearing loss (53.2%) [8]. Our survey found a smaller proportion of hearing loss was due to ear disease (7.5% left; 6.8% right vs 17.6%). The 2005-2006 survey found 26.8% of hearing loss was due to undetermined causes, and 60.0% due to non-infectious conditions. If we consider these causes together as acquired hearing loss (total of 86.8%), our study found a slightly higher proportion of hearing loss was due to acquired sensorineural hearing loss (91.6% left; 92.4% right). Differences may be due to the protocol used to assign causes or genuine differences in causes by region [43].

This survey provides useful information for planning services in Gao’an and provides a baseline for future studies in the county. In terms of service needs, over half of people needed diagnostic audiology and possible hearing aid fitting (54.0%). A smaller proportion needed wax removal (3.4%), surgical review (4.8%), or medication (2.0%). Only 0.4% of those in need had hearing aids. Very few people had previously sought care, the vast majority reported they did not feel the need. This may be due to lack of awareness about hearing loss and available treatments. At present, hearing aid fittings in the public system in Gao’an are provided only through hospitals in Nanchang. This study provides evidence that hearing aid services should be scaled up in Gao’an county and awareness raised. The prevalence of self-reported hypertension, smoking and diabetes were high – suggesting that primary and secondary prevention strategies are required for these conditions.

### Implications: survey protocol

There are some components of the RAHL methodology that need further attention. The analysis has reinforced some of the known challenges with collecting detailed information about the causes of hearing loss [22, 25, 45]. For simplicity, we did not try to specify a specific cause of sensorineural hearing loss, but instead categorised probable sensorineural hearing loss in to acquired and congenital. This decision was made for pragmatic reasons. The RAHL aims to provide information for planning purposes, and given the service need does not vary by underlying cause of sensorineural hearing loss we believe that grouping sensorineural hearing loss together in this way is appropriate. The risk factor analysis showed that age was significantly associated with hearing loss, suggesting that a large proportion of the acquired causes could be attributed to age related hearing loss or presbyacusis.

However, inconsistencies in cause assignment were identified. For example, when a participant had severe or profound loss and the cause was assigned as impacted wax. In this situation, the cause is unlikely to be impacted wax alone – an underlying sensorineural component likely exists in addition. These causes were recoded as mixed in the analysis (sensorineural hearing loss with wax impaction). The future intention with RAHL is to develop an automated analysis platform, and these types of inconsistencies may create barriers to this process. To improve consistency in cause assignment, further decision support within the mobile questionnaire will be developed for future surveys.

Further guidance is needed on how to assign the probable cause when there are two causes in the same ear. The RAAB survey takes the approach of assigning the cause that is most amenable to treatment [46]. This could be explored for RAHL. Due to the complexities of assigning cause at the level of the individual, we examined the causes in each ear separately. In future developments of the RAHL protocol, an improved method to assign causes could be developed using expert consensus (e.g. by ease of treatment, by better ear). Previous all-age surveys of hearing loss have provided scarce details on the methods of assigning cause in the individual despite presenting data in this way [43, 47]. Further attention into the most useful way to report data for planning purposes is also required.

To help overcome some of the challenges with cause assignment, risk factor analysis was conducted. This analysis found that only age, sex, malaria and literacy were associated with hearing loss. The utility of including an analysis of association with known risk factors in a survey report intended for planning is not clear. Recall bias and lack of health awareness may affect the responses. The specific questions used to ascertain data on risk factors should be reviewed in light of these findings. This analysis should be replicated in other settings before determining its value.

In future research, we will examine whether the addition of tympanometry may help with assigning the type, and reduce the proportion of unknown causes. Tympanometry may be useful in the diagnosis of OME, which can be more difficult to determine from otoscopy alone [48].

### Strengths and limitations

This survey has several strengths. A rigorous two-stage sampling procedure was used to select participants. The response rate was very high. Validated smartphone tools were used, for the first time in a population-based survey of hearing loss.

There are also some limitations with the study that should be taken in to account. Measuring SEP is important for understanding health equity. Previous work has recognised the difficulties in differentiating SEP based on assets in upper middle-income countries such as China, where the majority of households own key domestic goods [49]. For this field-test, an asset list was developed using previous questionnaires from China, including the China Living Standards survey [50]. The list was adjusted in the training period, based on local knowledge, and piloting. We found literacy (a proxy indicator of SEP) was associated with hearing loss, but the wealth indicator was not. Further evidence is required to understand how best to measure SEP in a rapid manner, in upper middle-income contexts such as China. A more nuanced asset-based measure may be required. The method of assessment of SEP in RAHL surveys should be standardised as far as possible, however specific questions are likely to vary based on context and income level.

## Conclusions

The RAHL survey methodology was developed to measure the prevalence and probable causes of hearing loss in population-based surveys in a low cost and rapid manner. Specifically, it aims to overcome the financial and logistical barriers of previous all-age surveys of hearing loss allowing data to be obtained quickly and utilised in district level planning. Field-testing of the RAHL survey in China confirmed that the protocol is feasible, and substantially faster than an all-age survey. The prevalence of moderate or greater hearing loss in the 50+ was high, increases with age, and decreases with degree. The prevalence found was also comparable to previous surveys conducted in China. The vast majority of people in the survey had hearing loss that was probable sensorineural or mixed in nature, and have the potential to benefit from hearing aids. Information gathered from this survey can be used to plan and advocate for improved ear and hearing care services at the county-level in Gao’an. Aspects of the survey that need further development include how causes could be better assigned, and how data should be reported in for planning purposes.

## Data Availability

The datasets used and/or analysed during the current study are available from the corresponding author on reasonable request.

## Appendix 1

**Table 8:** Algorithm for middle ear conditions/conductive causes of hearing loss

		Ear canal examination				Tympanic membrane examination					Middle ear
Condition	Pain reported	Ear canal inflammation	Occluding wax	Occluding foreign body	Discharge	Perforation	Light reflex visible	Malleus bone visible	Shape	Colour	Fluid present
Normal ear and normal hearing	No	No	No	No	No	No	Yes	Yes	TM shape normal	Pearly white	No
Acute otitis media (AOM)	Yes in ear canal	No	No	No	No	No	Yes/No	No	Bulging	Red	Yes or no
Otitis media with effusion	No	No	No	No	No	No	Yes or no	Yes	Retracted	Dull/Opaque	Yes or unclear
Chronic otitis media - wet	No	No	No	No	Yes for > 2 weeks	Yes	No or unable to see	Yes or no or unable to see	TM shape normal, bulging or irregular or unable to see	Pearly white or unable to see	No or unable to see
Impacted wax	No	No	Yes	No	No	Unable to see	Unable to see	Unable to see	Unable to see	Unable to see	Unable to see
Foreign body (FB)	No	No	No	Yes	No	Unable to see	Unable to see	Unable to see	Unable to see	Unable to see	Unable to see
Dry perforation	No	No	No	No	No	Yes	No	Yes or no	TM shape normal, budging or irregular	Pearly white	No
Otitis externa	Yes in ear canal	Yes	No	No	Yes or no (scanty discharge or scaling skin)	No or unable to see	Yes or unable to see	Yes or unable to see	Normal or unable to see	Normal or unable to see	No or unable to see

## Appendix 2

**Table 9:** Age and sex structure of persons aged 50 and above of the population of Jiangxi province (2010 census data) compared to the sample from Gao’an

	Male				Female				Total			
	Population		Sample		Population		Sample		Population		Sample	
	N	%	N	%	N	%	N	%	N	%	N	%
50–54	1,256,966	25.5	69	13.0	1,214,952	24.6	163	20.1	2,471,918	25.0	232	17.3
55–59	11,673,10	23.7	61	11.4	1,130,000	22.9	115	14.2	2,297,310	23.3	176	13.1
60–64	878,288	17.8	93	17.5	832,669	16.9	150	18.5	1,710,957	17.3	243	18.1
65–69	582,238	11.8	129	24.2	561,531	11.4	150	18.5	1,143,769	11.6	279	20.8
70–74	490,625	9.9	103	19.3	499,742	10.1	108	13.3	990,367	10.0	211	15.7
75–79	314,505	6.4	38	7.1	355,101	7.2	48	5.9	669,606	6.8	86	6.4
80–84	163,313	3.3	23	4.3	207,453	4.2	44	5.4	370,766	3.8	67	5.0
85–89	62,894	1.3	12	2.3	99,025	2.0	25	3.1	161,919	1.6	37	2.8
90+	17,104	0.3	5	0.9	34,770	0.7	8	1.0	51,874	0.5	13	1.0
Total	4,933,243	100	533	100	4,935,243	100	811	100	9,868,486	100	1344	100

## Appendix 3

**Table 10:** Proportion of hearing tests conducted in ambient noise above MPANLS (total = 1344)

	L	R
500	4.69%	4.47%
1000	1.71%	2.76%
2000	0.15%	0.22%
4000	0.15%	0.15%

## Abbreviations

AOM: Acute Otitis Media
CI: Confidence Interval
CSOM: Chronic Suppurative Otitis Media
dB: Decibel
DEFF: Design Effect
DP: Dry Perforation
ENT: Ear Nose and Throat
FB: Foreign Body
IOV: Interobserver Variation
IQR: Interquartile Range
IW: Impacted Wax
LMICs: Low and middle income countries
MPANL: Maximum Permissible Ambient Noise Level
ODK: Open Data Kit
OE: Otitis Externa
OME: Otitis Media with Effusion
OR: Odds Ratio
RAAB: Rapid Assessment of Avoidable Blindness
RAHL: Rapid Assessment of Hearing Loss
SEP: Socioeconomic Position
UHC: Universal Health Coverage
WHO: World Health Organisation
